# Commentary: Virtual Reality, Real Emotions: A Novel Analogue for the Assessment of Risk Factors of Post-traumatic Stress Disorder

**DOI:** 10.3389/fpsyg.2022.936930

**Published:** 2022-06-09

**Authors:** Zhongyu Shi

**Affiliations:** College of Creative Culture and Communication, Zhejiang Normal University, Jinhua, China

**Keywords:** PTSD, VR, TFP, induced PTSD, trauma film paradigm

With the progress of media technology, the image world has turned from a two-dimensional plane to a three-dimensional, and the sense of immersion/“presence” (Sanchez-Vives and Slater, 2005), which is closely related to body perception, has become the most controversial topic in the media era (Geng, 2022). From Merleau- Ponty's pioneering philosophical thinking to the present (Merleau-Ponty, 1965), the relationship between body and space as the core of digital media constitutes one of the most worth exploring trends of human-computer interaction (Evans and Rzeszewski, 2020). The trauma film paradigm was developed to show movies depicting traumatic events to non-clinical participants to trigger a stress response similar to the response to traumatic events in real life (Speisman et al., 1964). Trauma movies usually consist of several clips, such as the scenes of car accidents and interpersonal violence. It has been proved that watching such movies with various scenes can reliably induce similar symptoms (Weidmann et al., 2009; James et al., 2016). Although the TFP can act as an analog of PTSD, inducing involuntary invasion, the participants are still “outsiders,” unable to completely immerse themselves in the film scenes and emotionally separated from the events (Lazarus et al., 1965; Koriat et al., 1972).

As early as 2015, Pauline Dibbets and others creatively realized that VR showed different effects in trauma induction compared with TFP. Therefore, Dibbets and their colleagues recruited 43 college students and divided them into two groups, with an average age of 22.16 years and 23.45 years, respectively (Dibbets and Schulte-Ostermann, 2015). They compared the VR paradigm with the traditional traumatic film (TFP) on post-traumatic stress disorder (PTSD).

It is assumed that compared with TFP, PTSD caused by VR can make people immerse deeper, cause negative emotions and ruminate more strongly. However, the research results are quite the opposite. Compared with TFP, VR does not lead to more extreme changes in negative emotions or more (painful) invasions (Dibbets and Schulte-Ostermann, 2015). Such results are not typical. Generally speaking, the psychological and physiological activation in VR is generally high (Schweizer et al., 2018). Although the results need to be discussed, this experiment finally innovatively inspired us. The research contents of Dibbets are presented in tabular form, as shown in Table 1.

**Table 1:** Comparison of research results.

**Study target**	**Conclusion**	**The limitation of this study**	**Causes of limitations**	**Subsequent research**
Negative Emotion and Invasion Frequency	Both methods can induce traumatic memory, even if VR is not as good as TFP.	VR trauma induction time is short and invasion frequency is low.	To ensure that long-term PTSD symptoms are not induced.	Used the VR paradigm to detect avoidance behaviors in different people and environments specifically (Dibbets et al., 2021)
Risk factor 1: Imagination ability	Higher imagination coincides with more virtual reality conditions, higher ability leads to more intrusions (VR conditions), and higher ability is related to less invasion pain (TFP conditions).	There are fewer participants in each case.	This study is the first time used PTSD as an induction method.	Used the VR paradigm to induce intrusive emotions and avoidant behaviors, They found that the VR paradigm increased negative emotions and heart rate, decreased positive emotions and heart rate variability, and observed the symptoms that caused stress (Dibbets, 2020)
Risk factor 2: Trait anxiety	None	Participants are non-clinical people, so it isn't easy to popularize the results to clinical people.	For humanitarian reasons, we cannot induce negative emotions in clinical people.	
Risk factor 3: Depression	None	Questionnaire related to risk factors has a limited range of changes.	There is no pre-selected experimental participant.	

Researchers are convinced that there are differences between TFP and VR paradigms. Generally speaking, VR quantifies the image scene into visible differences such as the number of details of physical injuries, the impact contrast of color differences, and so forth, which has higher imageability and can more effectively induce traumatic memory. TFP generally uses the lens perspective and close-up scenes of specific movie narratives, lacking subjective perspective switching ability and imagination space. Nevertheless, the actual research shows a contrary situation. The film paradigm seems to trigger a more robust stress response, which may be more similar to real-life trauma (Hilberdink et al., 2022). Moreover, the types of trauma (body, sex, traffic, disgust) caused by the themes of different films have different direct subjective and physiological responses (Arnaudova and Hagenaars, 2017). The film paradigm is an invasive memory caused by psychological trauma (Holmes and Bourne, 2008), even beyond the perspective of first-hand experience (James et al., 2016).

As this research is the first to use VR for trauma induction, it is limited by many conditions. For clinical and non-clinical people, different generalization mechanisms are individualized due to fear and anxiety (Kenntner-Mabiala et al., 2008) non-clinical people were taken as the experimental subjects in the experiment.

After that, the paradigm of PTSD induced by a series of movies and VR clips has been gradually improved, as shown in Table 1. The use of psychometrics can complement the paradigm to track physiological response and emotional regulation changes during PTSD (Yang et al., 2021).

In addition to VR being used as a way to induce traumatic memory for the first time, a series of ways to intervene in psychotherapy using VR technology has been gradually improved (Andreatta et al., 2010; Ready et al., 2010; Rizzo et al., 2011). For example, there are studies on VR cognitive rehabilitation training for clinical people (Jahn et al., 2021). A recent qualitative study emphasizes that VR in art therapy can bring positive experiences, such as positive emotions, games, and exploration (Hacmun et al., 2018, 2021; Kothgassner et al., 2019; Kaimal et al., 2020).

Although this study cannot prove that VR can induce PTSD more than traditional trauma movies, Pauline Dibbets et al.' s pioneering experiments prove that TFP and VR seem to be promising psychological intervention technology that deserves our continued exploration. Especially compared with the computer screen, VR has been proved to be more immersive (Visch et al., 2010; Ding et al., 2018; Jones, 2019; Pallavicini et al., 2019), and people can immerse themselves in a virtual environment and interact with it (Riva et al., 2007). VR contributes to a pleasant wake-up state because it brings a novel, intense and sensational experience (Bartsch et al., 2006; Bartsch and Viehoff, 2010), VR can be developed as an emotional medium to induce users' specific emotions. Factors such as hardware and software costs of VR should also be considered. Fortunately, with the growth of the VR commercial consumer market, we can see that the accessibility of VR software has increased, and the prices of VR equipment have decreased (Mishkind et al., 2017; Norr et al., 2018). On all accounts, using VR mobilizes the embodied perception of participants and highlights the importance of the body as a direct medium for feeling and contacting the external world (Human body perception from the inside out: Advances in visual cognition., 2006; Harris et al., 2015; Zahiu, 2020). Breaking through the previous mode of psychological intervention with visual media, Dibbets and his colleagues provided a convincing example of this kind of research and the future direction to explore.

## Author Contributions

The author confirms being the sole contributor of this work and has approved it for publication.

## Funding

This work was supported by Zhejiang Normal University.

## Conflict of Interest

The author declares that the research was conducted in the absence of any commercial or financial relationships that could be construed as a potential conflict of interest.

## Publisher's Note

All claims expressed in this article are solely those of the authors and do not necessarily represent those of their affiliated organizations, or those of the publisher, the editors and the reviewers. Any product that may be evaluated in this article, or claim that may be made by its manufacturer, is not guaranteed or endorsed by the publisher.
